# Haematoma block: a safe method for pre-surgical reduction of distal radius fractures

**DOI:** 10.1186/s13018-020-01819-y

**Published:** 2020-08-26

**Authors:** Tazio Maleitzke, Fabian Plachel, Florian Nima Fleckenstein, Florian Wichlas, Serafeim Tsitsilonis

**Affiliations:** 1 Center for Musculoskeletal Surgery, Charité - Universitätsmedizin Berlin, Augustenburger Platz 1, 13353 Berlin, Germany; 2 Department of Diagnostic and Interventional Radiology, Charité - Universitätsmedizin Berlin, Berlin, Germany; 3 Clinic for Orthopaedics and Traumatology, Uniklinikum Salzburg, Salzburg, Austria; 4 Julius Wolff Institute, Charité - Universitätsmedizin Berlin, Berlin, Germany; 5 Berlin Institute of Health, Berlin, Germany

**Keywords:** Distal radius fracture, Haematoma block, Local anaesthetic, Closed reduction

## Abstract

**Background:**

The haematoma block (HB) has been successfully used to minimise pain prior to otherwise painful closed reduction manoeuvres for distal radius fractures. However, the invasive manner of the procedure, which technically produces an open fracture, still raises the question, whether HBs increase the risk of infection compared to conventional intravenous analgesia (IA). The purpose of this study was to assess complication rates and safety of the HB procedure for the closed reduction of surgically treated distal radius fractures.

**Methods:**

We included 176 distal radius fractures in 170 patients in a retrospective mono-centric study, who underwent closed reduction and casting followed by definitive surgical care over a period of two years. Patients either received a HB or IA before closed reduction and were evaluated for minor and major complications over a follow-up period of four years.

**Results:**

Overall, 42 distal radius fractures were treated with a HB (23.9%) and 134 with IA (76.1%) before closed reduction. There were a single major (2.3%) and eight minor (19%) complications observed in the HB group compared to two major (1.4%) and 24 minor (17.9%) complications in the IA group. No significant differences were identified between the two groups. Sex and type of fracture had no effect on complication rates, however, younger patients experienced higher complication rates in comparison to older ones (*p* = 0.035).

**Conclusion:**

According to our data, the apprehensions that clinicians may have of creating open fractures through HB procedures, are unnecessary and may be abandoned confidently.

## Background

Distal radius fractures are the most common extremity bone fractures and account for approximately 18 and 25% of all elderly and youth fractures, respectively. The prevalence of distal radius fractures has been growing consistently in recent years and approximately 1.5% of accident and emergency department (A&E) visits are due to distal radius fractures [1–3]. In 1986, Knirk and Jupiter pointed out the importance of an anatomical reduction in their often cited article entitled ‘intra-articular fractures of the distal end of the radius in young adults’ [4]. If a closed reduction is possible, adequate analgesia is crucial for the patient’s comfort, as well as for radiographic post-reduction results, including dorsal tilt, radial inclination and ulnar variance [5, 6]. Common types of currently used analgesia or anaesthesia include the haematoma block (HB), the Bier’s block, intravenous analgesia (IA) and general anaesthesia.

As an alternative to time- and staff-consuming general anaesthesia, a transcutaneous injection of local anaesthetic into the fracture haematoma, also known as a HB, is commonly used in A&Es for closed reduction manoeuvres. Although HBs are widely used to alleviate pain during closed reductions of distal radius fractures, some A&E physicians remain apprehensive about performing them. Since the needle used to instil the local anaesthetic creates a passage between the outside world and the internal fracture environment, this passage could in theory result in introducing an infection to the fracture site [7–9].

Whilst there have been studies reporting adverse events associated with HBs, namely, osteomyelitis and seizures [10–12], other studies could not confirm such complications [6, 13, 14].

The aim of this study was to compare complication rates of patients receiving either HB or IA for the reduction of distal radius fractures, that were later treated surgically in a university hospital level-I trauma centre. We hypothesised that the resource-saving HB provides safe analgesia during closed reduction manoeuvres for distal radius fractures that receive surgical care at a later stage.

## Methods

### Study population

We performed an electronic search of our A&E database for all patients over 18 years of age, who were diagnosed with a distal radius fracture over a two-year period (2012-2013). The search revealed 489 cases. To evaluate the safety of the HB for surgically treated patients and to ensure a homogenous study population, only patients who underwent surgery were included. We further excluded all patients who were initially treated at an outside facility or who did not undergo closed reduction manoeuvres in our A&E. Patients who were admitted straight to the emergency operating theatre and patients who were treated conservatively by casting alone were also excluded. The decision for surgical treatment was based on fracture instability and on the patient’s individual request. After exclusion, our study population consisted of 176 distal radius fractures in 170 patients, of which 42 fractures were treated with a HB (23.9%) (10 mL prilocain 2% (20 mg/mL), Astra Zeneca GmbH, Wedel, Germany) and 134 with IA (76.1%) (7.5-15 mg of piritramide, Hameln Pharma Plus GmbH, Hameln, Germany) before closed reduction and casting in our A&E. The mean age at the time of injury was 59.9 ± 18.3 years (range, 20 to 94.2 years). Women accounted for 119 cases (67.6%) and men for 57 cases (32.4%). The mean age in the HB group was 57.6 ± 17.9 years and 60.6 ± 18.5 years in the IA group. Demographic data of included patients are shown in Table 1.

**Table 1:** Demographic data of included patients divided by analgesic method

	HB	IA
Fractures, *n*	42	134
Fractures, %	23.9	76.1
Female/male gender, *n*	29/13	90/44
Female/male gender, %	69/31	67.2/32.8
Age, mean ± SD, years	57.6 ± 17.9	60.6 ± 18.5

*HB* haematoma block, *IA* intravenous analgesia

### Patient care

Patients presenting to our A&E with acute wrist pain following a trauma and a suspected distal radius fracture were treated with a preliminary splint for the wrist. The forearm was gently cooled with an ice pack and placed in an elevated position. X-rays of the injured wrist were promptly taken in two planes. Additional examinations of the neuro-vascular bundle of the forearm, wrist, and hand were performed and repeated if needed.

After the radiological diagnosis of a distal radius fracture, fractures were classified according to the AO/OTA system [15]. Seventy distal radius fractures were classified as type ‘A’ (39.8%), 22 were classified as type ‘B’ (12.5%) and 76 were classified as type ‘C’ (43.2%). For eight fractures no pre-procedural X-rays were available (4.5%). The indication of surgical treatment for distal radius fractures was based on instability and the patient’s individual wish.

### Haematoma block

**Fig. 1:**
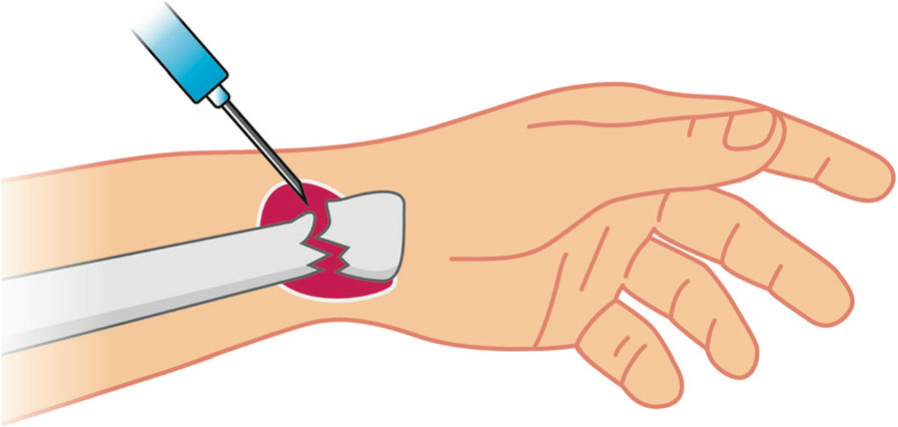
Schematic of the HB applied to a left distal radius fracture (HB, haematoma block)

For the HB, 10 mL of local anaesthetic, prilocain 2% (20 mg/mL), was drawn up into a syringe and the fracture site was identified by palpation of the dorsal wrist. The dorsal wrist was then cleansed with a disinfecting solution and draped in a sterile manner. The needle was inserted transcutaneously into the fracture site at a 30° angle, pointing from proximal to distal. Following our A&E standard operating procedure protocol, the needle’s correct location was confirmed by the use of a C-arm image intensifier and fracture haematoma was aspirated. 5-10 mL of prilocain was then injected into the region of the superficial sensory branch of the radial nerve and into the fracture haematoma itself [5] (Fig. 1).

**Fig. 2:**
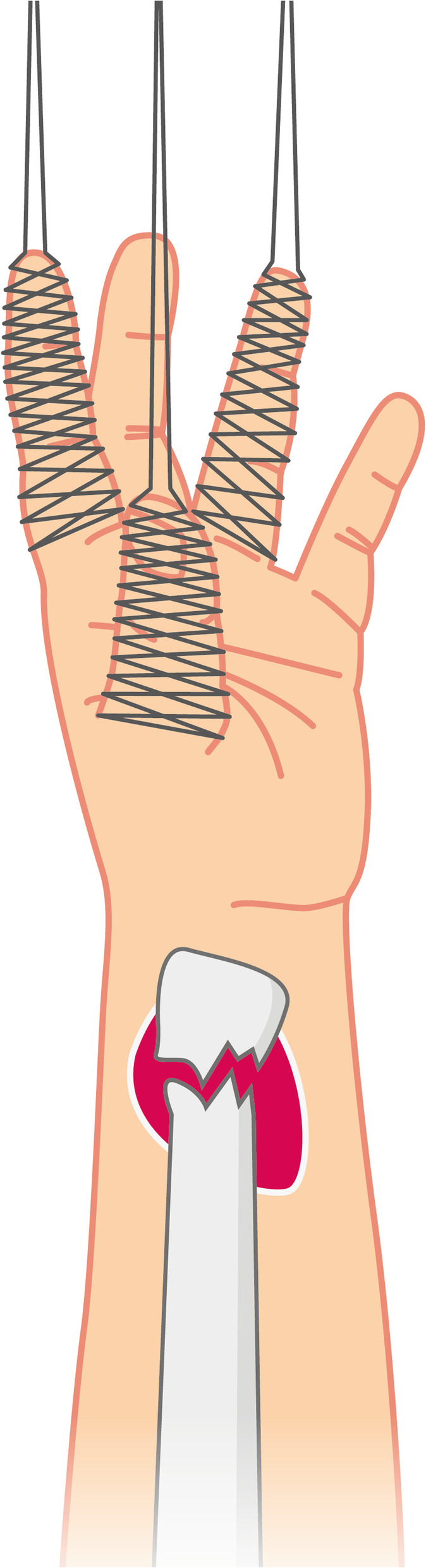
Schematic of Chinese finger-trap-traction applied to a left distal radius fracture

After 5-10 min, reduction was performed by Chinese finger-trap-traction with the help of a C-arm image intensifier. Chinese finger-trap-traction follows the principle of longitudinal traction and countertraction. Fingers of the affected forearm are fixed at the ceiling by finger-traps, creating traction (Fig. 2). Countertraction is gained through perpendicular angulation of the elbow and additional weights (2-5 kg) on the upper arm. Manual reduction with the help of a C-arm image intensifier was performed after maintaining traction for 15 min to reduce muscle tension. This step can also be performed without Chinese finger-trap-traction, yet this manoeuvre facilitates the reduction, especially in severely displaced fractures where the fracture gap may not be easily reached with the needle and the physician is on her/his own.

After closed reduction, a forearm plaster of Paris was applied, and post-reduction X-rays were obtained to confirm alignment. Relevant radiological parameters for reduction included dorsal tilt, radial inclination and ulnar variance [5].

### Surgical procedure

All 176 cases underwent definitive surgical treatment after reduction, casting and swelling control. An open reduction and internal fixation (ORIF) by plate osteosynthesis was performed in 174 cases (98.9%), whilst two cases (1.1%) received skeletal stabilization by external fixation.

### Complication analysis

Every complication over a period of four years postoperatively was included in the analysis. Major complications were defined as complex regional pain syndrome (CRPS) and local infection (Table 2). Minor complications were defined as tendinitis, sensory deficit, motor deficit, loss of reduction, carpal tunnel syndrome, neuralgia, keloid formation and postoperative ganglion (Table 3).

**Table 2:** Major complications divided by analgesic method

	HB, *n* = 1	IA, *n* = 2
CRPS	0	1
Local infection	1	1

*HB* haematoma block, *IA* intravenous analgesia

**Table 3:** Minor complications divided by analgesic method

	HB, *n* = 8	IA, *n* = 24
Tendinitis	2	3
Sensory deficit	1	3
Motor deficit	3	6
Loss of reduction	1	3
Carpal tunnel syndrome	1	6
Neuralgia	0	1
Keloid formation	0	1
Postoperative ganglion	0	1

*HB* haematoma block, *IA* intravenous analgesia

### Statistics

All statistical analyses were conducted using IBM SPSS Statistics Software Version 21. All values were described using descriptive statistics (mean, standard deviation (SD), minimum and maximum). The Kolmogorov-Smirnov test was used to test for normal distribution. To compare normally distributed data between the HB and the IA group, we used the independent *t* test. Furthermore, relationships between categorical variables were determined by the chi-square test. The Cramer’s *V* coefficient (CVC) was used to measure the strength of association between two categorical variables. Values close to 0 describe a weak association, values close to 1 describe a strong association. Analyses were performed with two-tailed *p* values and the alpha level was set at 0.05.

### Graphics

All figures were created using Adobe Illustrator Creative Cloud.

## Results

Overall, 35 (19.9%) complications were recorded, all of which appeared after definitive surgical treatment. Major complications were observed in three cases (1.7%). There was no significant difference in the distribution of major complications between the HB and IA group, as shown in Table 2 (CVC = 0.029, *p* = 0.698).

A single case (2.3%) of local infection was observed in the HB group. The patient subsequently underwent revision surgery and made a full recovery.

In the IA group, one case of local infection, as well as one case of CRPS (1.4%) were reported.

The patient with CRPS in the IA group had all surgical implants removed after fracture consolidation and recovered fully. The patient suffering from a local infection in the IA group was lost to follow-up before further treatment.

Minor complications were observed in 32 cases overall (18.5%). Eight cases in the HB group (19%) and 24 cases in the IA group (17.9%) showed minor complications, yet without any significant difference between groups (CVC = 0.013, *p* = 0.868). Minor complications included tendinitis (*n* = 2 in the HB group, *n* = 3 in the IA group), sensory deficit (*n* = 1 in the HB group, *n* = 3 in the IA group), motor deficit (*n* = 3 in the HB group, *n* = 6 in the IA group), secondary loss of reduction (*n* = 1 in the HB group, *n* = 3 in the IA group), carpal tunnel syndrome (*n* = 1 in the HB group, *n* = 6 in the IA group), neuralgia (*n* = 1 in the IA group), keloid formation/hypertrophic tissue (*n* = 1 in the IA group), postoperative ganglion (*n* = 1 in the IA group). The distribution of minor complications between groups is shown in Table 3.

Whilst gender had no effect on major (CVC = 0.091, *p* = 0.227) or minor (CVC = 0.114, *p* = 0.129) complication rates, age demonstrated a disparity. Overall complication rates were significantly more common in younger patients (mean age of 54.0 ± 16.8 years) when compared to their elder counterparts (mean age of 61.3 ± 18.4 years) (*p* = 0.035). Age had however no effect on the type of analgesia chosen (*p* = 0.358). Mean age in the HB group was 57.6 ± 17.9 years and 60.6 ± 18.5 years in the IA group. The type of fracture had also no effect on major (CVC = 0.067, *p* = 0.684) or minor complications (CVC = 0.043, *p* = 0.857) or the type of analgesia chosen (CVC = 0.136, *p* = 0.211).

## Discussion

The question of whether the HB is associated with higher complication rates than IA is still debated amongst A&E professionals today. The apprehension of transforming a closed fracture into an open fracture equivalent dates back to the 1980s, when R.D. Case amongst others described the theoretical risk of introducing an infection into the fracture site through the HB procedure [7–9].

Our findings show no difference in complication rates between HB- and IA-treated patients. Additionally, we were able to show that sex and type of fracture had no effect on minor, major or overall complications.

In 1991, Johnson and Noffsinger posed the question: ‘Haematoma block of distal forearm fractures. Is it safe?’ The prospective study found no signs of infection in patients treated with HBs. In their study the HB was compared to general anaesthesia and intravenous regional anaesthesia [16]. In the years to follow, the HB rose in popularity amongst UK A&Es from 7% in 1989 to 33% in 1994, whereas the use of general anaesthesia for the closed reduction of distal radius fractures declined from 44 to 24% during the same time period. Main reasons named for the more frequent use of HBs in A&Es were the more efficient patient flow and reduced costs when compared to the more time-consuming and expensive general anaesthesia [7].

More publications followed that proved the efficacy of the HB and that demonstrated its greater pain control and higher rates of patient acceptance compared to other anaesthetic techniques [9, 13, 14].

In this study, we further demonstrate that patients suffering complications after distal radius fractures were significantly younger than those without complications. This result was independent of the type of analgesic method.

Interestingly, a prospective cohort study by Chung et al. found that increased age is a predictor for worse long-term outcomes, when measured one year after the surgical treatment of distal radius fractures, using volar locking plate systems [17]. These contradicting results may be due to the fact that our patients were in general of older age and that the mean age of our patients suffering complications was rather close to that of our patients without complications (54.0 ± 16.8 years versus 61.3 ± 18.4 years). We therefore suspect this finding to be attributed to chance, especially because there was no significant age difference observed when major complications were analysed independently.

A recent study from 2016 showed that the HB proved to be especially effective in the elderly population. The HB group had therefore not only a significantly lower pain intensity compared to the general anaesthesia group during closed reductions but also a significantly lower duration of manipulation and a shorter time to discharge, which may be beneficial due to inherent comorbidities and higher rates of dementia [14]. Similar data were gathered for the paediatric patient collective, where the length of stay in the A&E was also significantly shorter in the HB group in comparison to the procedural sedation group [18].

As with every invasive procedure, the HB method bears risks, which should be carefully evaluated and discussed with the patient prior to the intervention. Although our data did not show any cases of systemic neurological reactions, there have been case reports of seizures following HB procedures [11, 12]. The most probable cause for these seizures was an intravenous injection of the local anaesthetic. Aspiration of blood is usually a sign for intravascular penetration, and no injection should be performed in that case. Yet, aspiration of blood from a fracture haematoma is also a sign of a correct intra-fragmentary position of the needle through which the local anaesthetic is administered. Therefore, a C-arm image intensifier may be used in every HB procedure to reassure the physician of the needle’s correct position during the administration of the local anaesthetic. As there is only a small number of case reports on systemic effects of local anaesthetic following HB procedures, it is not a likely complication in daily practice, yet worth noting. Meinig et al. showed that plasma levels of lidocain after HB procedures were well below the toxic threshold when administered correctly and therefore systemic side effects of correctly administered HBs are unlikely [19].

Our data show two cases of local infections post-operatively. One case was observed in the HB and one in the IA group. The patient in the HB group did not show any signs of osteomyelitis and recovered fully after a single revision surgery. Unfortunately, we cannot make any assumptions about the patient in the IA group, as he was lost to follow-up before any further treatment. Posttraumatic osteomyelitis cases after closed distal radius fractures have been reported previously [20, 21], yet cases of osteomyelitis after distal radius fractures, which were reduced using a HB are rare. However, one case of a *Staphylococcus aureus* osteomyelitis after HB treatment was reported in the UK in 2002 [10].

Bearing these findings in mind, our aim was to assess the safety of the HB before the closed reduction of distal radius fractures in the A&E of a university hospital level-I trauma centre, treating between approx. 60000 and 65000 patients annually in 2012 and 2013. Proving our hypothesis, the HB group did not show higher major or minor complication rates in comparison to the IA group. Complications observed in the HB group also appeared in the IA group with a comparable incidence.

Complications like CRPS and local infections have been described as common complications after plate osteosynthesis treatment for distal radius fractures, regardless of the method of pre-procedural analgesia [22]. The same applies to tendinitis, sensory and motor deficits, secondary dislocation, carpal tunnel syndrome, neuralgia and keloid formation, which were all reported to occur as complications after sustained distal radius fractures, independent of performed HBs [23–25]. It may therefore be that reported complications were due to the surgical intervention itself, rather than to the pre-operative HB procedure. However, since there was no difference in the occurrence of complications in either group, it can be stated that the HB is a safe method for the pre-surgical reduction of distal radius fractures. Limitations of this study were its mono-centric and retrospective character.

## Conclusions

We conclude that the HB is a safe method of administering analgesia during closed reductions of distal radius fractures in A&Es. The method should be preferred over IA if A&E staff are trained in the procedure and sterile conditions can be assured.

According to the findings of our study, the apprehension that physicians may have of transforming a closed distal radius fracture into an open distal radius fracture with higher complication rates cannot be supported and may confidently be abandoned.

## Data Availability

The datasets used and analysed during the current study are available from the corresponding author on reasonable request.

## Abbreviations

A&E: Accident and emergency department
CRPS: Complex regional pain syndrome
CVC: Cramer’s *V* coefficient
HB: Haematoma block
IA: Intravenous analgesia
ORIF: Open reduction internal fixation
SD: Standard deviation
